# Effects of Exercise on Gut Microbiota of Adults: A Systematic Review and Meta-Analysis

**DOI:** 10.3390/nu16071070

**Published:** 2024-04-05

**Authors:** Leizi Min, Alimjan Ablitip, Rui Wang, Torquati Luciana, Mengxian Wei, Xindong Ma

**Affiliations:** 1 Division of Sports Science and Physical Education, Tsinghua University, Beijing 100084, China; minlz23@mails.tsinghua.edu.cn (L.M.); almjablt22@mails.tsinghua.edu.cn (A.A.);; 2 Department of Public Health and Sport Sciences, Medical School, University of Exeter, Exeter EX1 2HZ, UK; l.torquati@exeter.ac.uk

**Keywords:** exercise, physical activity, human, gut microbiota, systematic review, meta-analysis

## Abstract

Background: The equilibrium between gut microbiota (GM) and the host plays a pivotal role in maintaining overall health, influencing various physiological and metabolic functions. Emerging research suggests that exercise modulates the abundance and functionality of gut bacteria, yet the comprehensive effects on GM diversity remain to be synthesized. Objectives and Design: The study aims to quantitatively examine the effect of exercise on the diversity of gut microbiota of adults using a systemic review and meta-analysis approach. Methods: PubMed, Ebsco, Embase, Web of Science, Cochrane Central Register of Controlled Trials, the China National Knowledge Infrastructure, and Wanfang Data were searched from their inception to September 2023. Exercise intervention studies with a control group that describe and compare the composition of GM in adults, using 16S rRNA gene sequencing, were included in this meta-analysis. Results: A total of 25 studies were included in this meta-analysis with a total of 1044 participants. Based on a fixed-effects model [Chi^2^ = 29.40, *df* = 20 (*p* = 0.08); *I*^2^ = 32%], the pooled analysis showed that compared with the control group, exercise intervention can significantly increase the alpha diversity of adult GM, using the Shannon index as an example [WMD = 0.05, 95% CI (0.00, 0.09); Z = 1.99 (*p* = 0.05)]. In addition, exercise interventions were found to significantly alter GM, notably decreasing Bacteroidetes and increasing Firmicutes, indicating a shift in the Firmicutes/Bacteroidetes ratio. The subgroup analysis indicates that females and older adults appear to exhibit more significant changes in the Shannon Index and observed OTUs. Conclusions: Exercise may be a promising way to improve GM in adults. In particular, the Shannon index was significantly increased after exercise. Distinct responses in GM diversity to exercise interventions based on gender and age implicated that more research was needed.

## 1. Introduction

There are approximately 10 trillion bacteria in the human intestinal tracts, which is approximately 10 times the total number of human cells [1], causing the intestinal microflora to be regarded as the “second genome” of the human body. The development of these microbial communities begins at the early stage of life, when microorganisms colonize the human body during infancy [2], and the composition of bacteria in the gut can be influenced by many aspects, including genetics, age, environment, diet, and lifestyle [3]. Different factors in this early life stage, such as type of birth, breastfeeding, and exposure to antibiotics, result in great differences in the composition and quantity of intestinal microflora among different people [4]. Under normal circumstances, all kinds of microorganisms maintain symbiotic or antagonistic relationships in the gut and together form a dynamic and balanced micro-ecosystem [5], which is essential for the integrity of the gut barrier and the normal function of the immune system [3]. Thus, when the diversity and balance of the gut microflora are disturbed, the risk of inflammatory diseases is greatly increased [6]. There are great differences in the composition and quantity of intestinal microflora among different people, but the structure will remain relatively stable. In recent years, advancements in high-throughput sequencing technologies have significantly enhanced human microbiome studies, facilitating the comparison of microbial diversity among different populations, particularly in the context of varying lifestyles and diets. Alpha diversity indices, such as Chao1, Simpson, Shannon, and Richness, are commonly employed to characterize the gut microbiota [7]. In this study, Chao1 and observed OTUs are utilized to assess the community richness of the gut microbiota, while the Shannon and Simpson indices are applied to evaluate its community diversity. Employing these diversity indices allows for a more comprehensive and nuanced exploration of how exercise influences gut microbiota diversity, providing insights into the multifaceted interactions between lifestyle factors and microbial communities.

There is a dynamic balance between gut microbiota (GM) and the host, which can maintain the normal physiological and metabolic functions of the body. However, different external and internal factors may affect GM composition and function [8]. Changes in health habits and dietary structure, exercise, and the use of antibiotics, as well as external microorganisms, affect intestinal flora [3,6,9,10]. More recently, exercise has emerged as an important factor in shaping GM composition in adults. A large body of evidence supports the role of exercise in effectively preventing and treating chronic diseases such as obesity, hypertension, depression, diabetes, respiratory diseases, and cardiovascular diseases [11]. As these diseases have also been associated with specific GM composition and function [12], exercise may be more closely related to intestinal flora than previously thought.

Recent studies have shown that exercise can shift the abundance and function of some gut-representative bacteria [13]. An early study on intestinal flora showed that the abundance and diversity of intestinal flora, as well as its metabolic functions, were significantly higher in professional rugby players compared to the general population of the same age and body mass index [14]. Another study on people with type 2 diabetes showed that the intensity of exercise also played a role in shaping the GM. Compared to high-intensity interval training, 8-week aerobic training resulted in a higher abundance of *Bifidobacterium*, *Akkermansia municiphila*, and butyrate-producers *Lachnospira eligens*, *Enterococcus* spp., and *Clostridium* Cluster IV [15]. However, there are also inconsistent results, with studies showing that high-intensity vigorous exercise can result in an imbalance of intestinal microflora and increased markers of inflammation [16]. The mixed results of exercise’s effect on intestinal flora may be related to the differences between studies in terms of the type of exercise (intensity, duration) and participants’ ages, genders, and health statuses. While previous systematic reviews have positively concluded that exercise has a positive overall effect on health and intestinal ecology, the best type, frequency, intensity, and time of exercise still need to be explored. To the best of our knowledge, none of the previously published reviews have provided a quantitative meta-analysis based on experimental studies to provide the overall effect size of exercise on GM composition and function.

Therefore, the aim of this study was to evaluate the effects of exercise on GM diversity (alpha and beta diversity indexes). Our secondary objectives were to summarize the effects of exercise on specific taxa abundance (i.e., Actinobacteria, Bacteroidetes, Firmicutes, etc.).

## 2. Methods

### 2.1. Protocol and Registration

This systematic review with a meta-analysis was registered in the International Prospective Register of Systematic Reviews (PROSPERO) trial registry (CRD42022382630). Additionally, the general guidelines of the Preferred Reporting Items for Systematic Reviews and Meta-Analysis (PRISMA) Statement were followed.

### 2.2. Literature Search Methods

PubMed, Ebsco, Embase, Web of Science, Cochrane Central Register of Controlled Trials, the China National Knowledge Infrastructure, and Wanfang Data were searched from their inception to September 2023. The search strategy combined the 2 groups of relevant terms: (1) “Gut Flora”[Mesh] OR “gut flora”*[tw] OR ”gut microbiome “*[tw] OR “gut bacteria”*[tw] OR intestinal flora *[tw] OR intestinal microbiota *[tw]; (2) “Exercise”[Mesh] OR exercise*[tw] OR exercising[tw] OR “Exercise Therapy”[Mesh] OR “Physical Fitness”[Mesh] OR “Resistance Training”[Mesh] OR aerobic activit*[tw] OR physical activit*[tw] OR sport*[tw] OR walk*[tw] OR run*[tw] OR jog*[tw] OR swim*[tw] OR cycling[tw] OR bicycl*[tw] OR physical training*[tw] OR danc*[tw] OR Resistance[tw] OR pilates[tw] OR strength[tw].

### 2.3. Inclusion and Exclusion Criteria

English studies were included if they met all of the following criteria: (1) Participants: adults (18–75 years old), including athletes, healthy non-athletes, obese patients, and sedentary people, etc.; (2) intervention: any type of exercise, physical activity, or sport/training; (3) outcomes: study measured changes in GM measure using 16s rRNA sequencing, reported diversity indexes and/or abundance of GM taxa and/or functional analysis providing sufficient data to be extracted, and analyzed mean effects and 95% confidence intervals (CI); (4) study design: intervention studies with an appropriate control group (e.g., rest control, health education, or usual care control) or crossover studies allowing for the discernment of causal effects. However, comments, animal model subjects, conference abstracts, case reports, and reviews were excluded. We also excluded studies with incomplete outcome data or interventions that included a dietary component, supplements, probiotics, or antibiotic drugs.

### 2.4. Assessment of Quality

Two independent reviewers assessed the quality of the included studies using the risk of bias tool provided by the Cochrane Handbook for Systematic Reviews of Interventions. A total of 7 items were assessed for each study: selection bias (random sequence generation and allocation concealment), performance bias (blinding of participants and personnel), detection bias (blinding of outcome assessment), attrition bias (incomplete outcome data), reporting bias (selective reporting), and other bias. For each item, there are three potential bias judgments: “low risk”, “high risk”, or “unclear risk”. Disagreements between the reviewers were resolved by discussion, with the involvement of a third author where necessary.

### 2.5. Data Collection Process

The original data from all of the selected studies were extracted on a prespecified worksheet. For those without available original data, data were requested via E-mail from the authors. This included study characteristics (first author’s name, year of publication, etc.), participant characteristics (age, gender, number, etc.), details of interventions (type of exercise, frequency, intensity, duration, length, etc.), mean of pre- and post-exercise training primary, and secondary outcomes with standard deviation.

### 2.6. Statistical Analysis

We performed statistical analyses using Cochrane’s Review Manager software (V.5.4). Among them, the weighted mean difference (WMD) was used for continuous variables, and the confidence interval (CI) was 95% CI. In the case of large difference in values or different data units, the standardized mean difference (SMD) and its 95% CI were used as the effect quantity for analysis. The heterogeneity between studies was evaluated using the Q test and expressed by the statistical value of *I*^2^, so as to determine the fixed-effect model or random-effect model. If *I*^2^ > 50%, the random-effect model was used, and sensitivity analysis was carried out to identify the source of heterogeneity by excluding one study at the time. If *I*^2^ ≤ 50%, the fixed-effect model was used for analysis. For studies that only used graphs to represent data, we used the Y-axis and length of the histogram to estimate the mean and standard deviation (SDs). The mean value and SD value of the difference between the intervention group and the control group before and after the intervention were used to calculate the effect size (ES) of each included study. Funnel plots were used to evaluate publication bias.

## 3. Results

### 3.1. Study Selection

**Figure 1:**
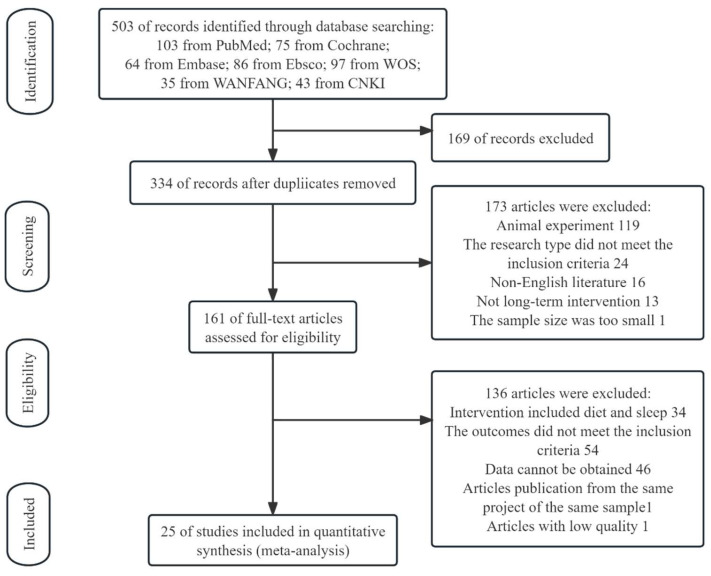
PRISMA flow diagram of the systematic review process.

At the end of the selection process, 503 articles were extracted, of which n = 97 were from Web of Science, n = 103 were from PubMed, n = 86 were from Ebsco, n = 64 were from Embase, n = 75 were from Cochrane Library, 35 were from WANFANG, and 43 were from CNKI. Each title and abstract were screened for relevance, removing review articles, comments, animal model subjects, conference abstracts, case reports, practical guidelines, books, and book chapters (n = 334). Thereafter, the screening was based on the assessment of the full text of the remaining 161 articles to verify their eligibility. Lastly, 25 research articles specifically focusing on the effect of physical activity or exercise on GM in healthy and unhealthy participants were included (Figure 1).

### 3.2. Characteristics of Studies

Table 1 summarizes the characteristics of the studies included, which were conducted between 2016 and 2023. The greatest numbers were performed in the United States, Spain, Australia, Ireland, Canada, and China, with one study each from the UK, New Zealand, Italy, Korea, Finland, Danish, Germany, Brazil, Slovakia, Netherlands, and Turkey. In total, this review pooled results from 1044 subjects, with sample sizes that ranged from 12 to 109 and participants’ ages that ranged from 16 to 70 years. The majority of the studies involved both genders; nine included only males and four examined females only. As to the intervention, researchers often used HIIT, MICT, aerobic exercise, or resistance training. Regarding the GM assessment, most classified the bacteria detected according to both phylum and genus. Few studies, 18 out of 25, included the level of species when classifying the bacteria detected. The V3–V4 or V4 region of the 16S rRNA gene were mostly amplified, one study selected V1–V2, V1–V3, V4–V5, or V5–V6, respectively, while three studies did not specify region. Most of the investigations were randomized controlled trials, and some were crossover studies. On the other hand, the period of five studies was eight weeks while one study followed patients for six weeks.

### 3.3. Quality of the Evidence

The quality of the studies is summarized in Supplementary Figures S1 and S2. The results showed a low risk of bias and only one article did not use blinding and another one selectively reported data. Overall, the quality of the included studies is relatively reliable.

### 3.4. Primary Outcomes

**Figure 2:**
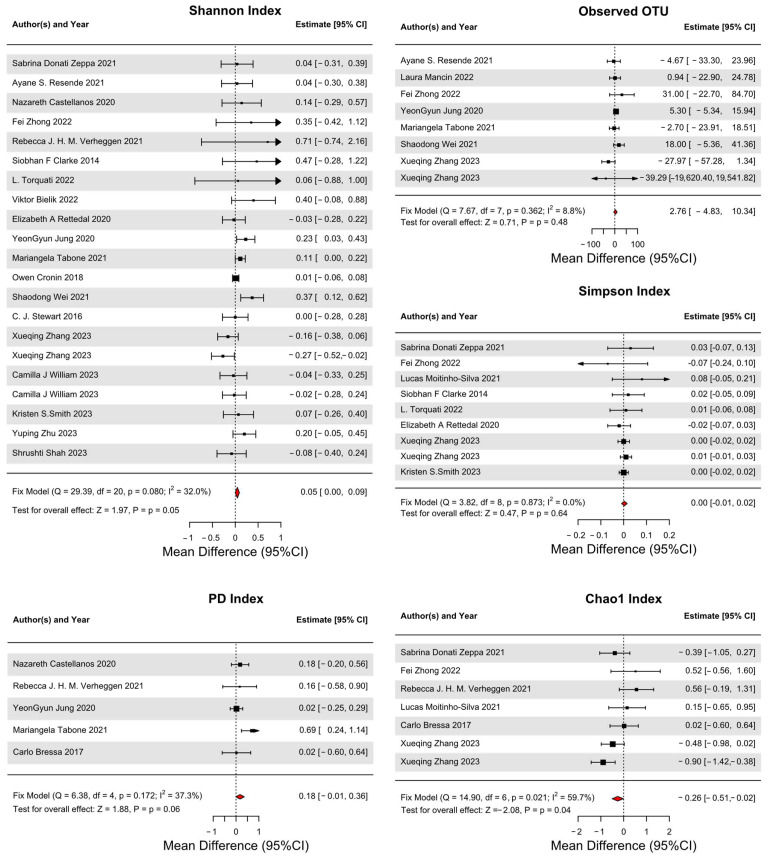
Forest plots of gut microbiota diversity in adults [14,15,17,18,19,20,21,22,23,24,25,26,27,28,30,31,32,33,34,36,38,39].

In this meta-analysis, we investigated the impact of exercise interventions on adult GM diversity, focusing on the Shannon, Simpson, Chao1 indices, and OTU (Figure 2 and Figures S3–S5). The analysis revealed a notable improvement in the Shannon Index under a fixed-effects model, indicating enhanced GM diversity following exercise intervention. The combined data exhibited a WMD of 0.047, within a 95% CI from 0.001 to 0.092, and a Z-value of 1.993 (*p* = 0.046), denoting a statistically significant impact.

Conversely, the Simpson Index results did not demonstrate statistical significance as the ES was 0.0025, with a 95% CI from −0.009 to 0.014, and a Z-value of 0.431 (*p* = 0.666), suggesting no significant exercise influence on this diversity measure. Similarly, the Chao1 Index, although specifically examined in some studies, did not show a significant standardized mean difference in the fixed-effects model, with a Z-value of 0.801 (*p* = 0.423), indicating variable effects of exercise on this diversity parameter across the sampled population. The analysis of observed OTUs yielded an ES of 2.759, with a 95% CI ranging from −4.827 to 10.345. Despite the broad range, the Z-value of 0.713 (*p* = 0.476) suggests that these changes in OTUs due to exercise were not statistically significant in our dataset.

The findings collectively suggest that while exercise can have a beneficial impact on GM diversity, as particularly evidenced by the Shannon Index of 19 studies, the relationship is not uniform across all measures of diversity, indicating that the effects of exercise are more likely reflected in evenness rather than richness. These results highlight the need for a nuanced understanding of how different types of exercise may selectively influence various aspects of GM. Future research should strive for a more comprehensive sample and employ consistent measurement methodologies to unravel the precise nature of how physical activity (PA) modulates GM diversity.

### 3.5. Secondary Outcomes

In this meta-analysis, we extended our investigation to the effects of exercise on specific bacterial phyla in GM, as well as the overall microbial diversity, as measured by the Phylogenetic Diversity (PD) index (Figure 2, Figures S6 and S7).

Our analysis of the PD index of five studies did not reveal significant changes attributed to exercise, presenting a marginal *p*-value of 0.063. This finding suggests that the influence of exercise on the broader microbial evolutionary diversity might be subtle. This result underscores the complexity of the interplay between PA and the GM ecosystem, hinting at intricate relationships influenced by individual biological variances.

Significant Changes in Specific Bacterial Groups:

Bacteroidetes: The analysis indicated a significant decrease in the abundance of Bacteroidetes, as evidenced by the negative ES of −0.29 and a statistically significant *p*-value of 0.013. This shift suggests that exercise interventions might lead to a reduction in this particular phylum, which is a key component of the GM. Bacteroidetes are known for their role in complex carbohydrate digestion and energy metabolism. The observed post-exercise decrease raises questions about the specific impact of PA on this group and its potential metabolic implications.

Firmicutes: In contrast, Firmicutes, another dominant GM phylum, showed a significant increase following exercise, with an ES of 1.486 and a highly significant *p*-value (*p* < 0.0001). This increase suggests that exercise interventions may favorably influence the abundance of Firmicutes in the gut. Firmicutes are involved in energy extraction and storage, and their increased presence could be associated with enhanced metabolic processes in the gut. The marked elevation observed in response to exercise highlights the beneficial role of PA in modulating this phylum, potentially contributing to better gut health and metabolic balance.

Given these results, exercise interventions are associated with a change in the Firmicutes/Bacteroidetes ratio (F/B ratio) within the GM, likely leading to an increase in this ratio. Since the F/B ratio is often considered a marker of gut health, with some studies linking higher ratios to obesity and metabolic disorders [28], the observed alteration could have implications for the metabolic profile and overall health of the individual. However, it is essential to interpret these findings with caution, as the clinical significance of F/B ratio changes remains a topic of ongoing research, and the relationship between this ratio and health outcomes is not yet fully understood.

Proteobacteria: Often associated with inflammatory conditions, the levels of Proteobacteria did not exhibit significant changes post-exercise intervention (*p* = 0.212). This lack of significant alteration underscores the multifaceted effects of exercise on GM, likely dependent on variables like exercise type, intensity, duration, and individual baseline GM composition.

Overall, our analysis indicates that exercise has a significant impact on certain bacterial populations in the GM, especially in the Bacteroidetes and Firmicutes phyla. However, these effects are not uniformly observed across all bacterial groups, highlighting the need for future research to explore these relationships in more detail. This research should consider individual variations, types of exercise, and the long-term effects of physical activity on GM composition to comprehensively understand the role of exercise in gut health.

### 3.6. Subgroup Analysis

**Figure 3:**
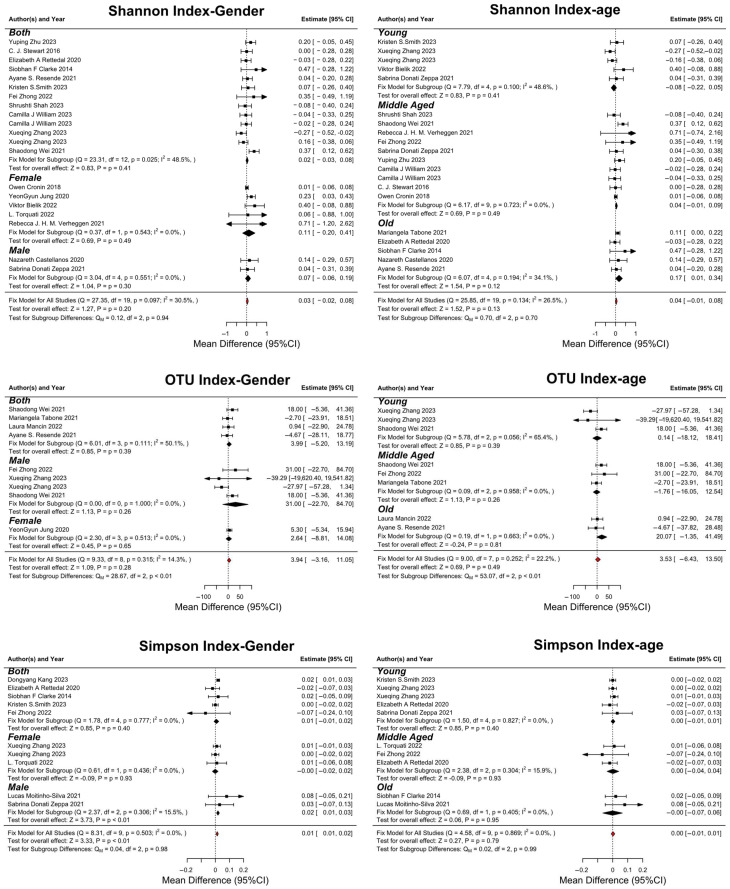
Forest plots of alpha diversity based on gender and age [14,15,17,18,19,20,21,22,23,24,25,26,27,28,30,31,33,34,36,37,38,39].

The subgroup analysis in our meta-analysis was conducted based on gender and age categories, focusing on three primary outcomes: the Shannon Index, Observed OTUs, and the Simpson Index (Figure 3 and Figures S3–S5).

Gender-Based Analysis:

The gender-based analysis revealed a mean difference in the Shannon Index of 0.019 for all participants, with a more pronounced effect in males (0.067) compared to females (0.106). This suggests that exercise may have a more substantial impact on GM diversity in females than males. For observed OTUs, the gender-based mean difference was more significant in females compared to males (2.637), indicating a potentially greater change in overall microbial richness in females following exercise. The gender-based mean difference in the Simpson Index was minimal (0.00251219) for both genders, indicating a less pronounced impact of exercise on this aspect of microbiota diversity.

Age-Based Analysis:

The age-based analysis of the Shannon Index showed a notable mean difference in older adults (0.266) compared to middle-aged (0.034) and younger individuals (−0.081), suggesting that older adults might experience greater benefits in terms of GM diversity from exercise. The mean difference in observed OTUs was highest in older adults (20.07), followed by middle-aged (−1.76) and younger individuals (0.14), again highlighting a more pronounced response in older adults. In the age-based analysis, the mean difference in the Simpson Index was relatively small across all age groups, with the most notable difference in older adults (−0.0004), identifying a less significant impact of exercise on this diversity measure across different age groups.

The subgroup analysis indicates distinct responses in GM diversity to exercise interventions based on gender and age. Particularly, females and older adults appear to exhibit more significant changes in the Shannon Index and observed OTUs, suggesting a potential age and gender-specific influence of exercise on GM. These findings highlight the importance of considering demographic factors when assessing the impact of exercise interventions on GM diversity. Further research is needed to explore these relationships and understand the underlying mechanisms driving these differences.

## 4. Discussion

### 4.1. Findings and Novelty of This Review

This review summarizes 25 studies involving the effects of exercise on the GM of professional players, college students, normal healthy adults, or chronic disease patients (obesity and diabetes). In brief, this review aimed at evaluating the effects of exercise on alpha diversity and beta diversity of GM and then summarizing the effects of exercise on specific flora. To the best of our knowledge, there is no quantitative meta-analysis based on 16S rRNA gene sequencing experimental studies focusing on the effects of exercise on GM. This study updates the previous published meta-analysis, including a greater number of studies and providing a stronger and more comprehensive overview of the present knowledge about the composition of the intestinal flora in adults with and without exercise. Furthermore, this meta-analysis pools the results of 25 studies with high-quality evidence and provides joint information on the relative abundance of bacteria belonging to specific phyla and genera in professional players, healthy physically active or inactive adults, and overweight or diabetes diagnosis people [40,41].

### 4.2. Changes in Microbial Diversity Index

The diversity index is usually regarded as the major indicator of human gut microbiome status. Here, we highlight a positive association between better gut microbiome diversity (Shannon index) and physical activity, suggesting that regular exercise might modulate microbial composition. This positive influence might be attributed to the modulation of exercise improving dysbiosis in the gut [42]. There are several hypotheses explaining potential mechanisms, including increasing production of intestinal immunoglobulin A, changes in the profile of bile acids, reducing serum lipopolysaccharide levels by suppressing Toll-like receptor 4 signaling pathways, and inducing hormetic effects of stress to stimulate beneficial adaptations of the intestinal barrier [43,44]. Simultaneously, the subgroup analysis was conducted based on gender and age categories. According to previous studies, some bacteria associated with various attributes suggestive of a healthy host metabolism were enriched more easily in females than males [40]. The age-associated human GM features were possibly related to the higher bacterial gut diversity in old adults, and bacterial diversity was proven to increase with age [41].

We did not find a significant change in the alpha (Simpson, Chao1 indices, and OTU), or beta diversity indices, which might depend on differences in exercise intensity and performing duration [45]. As the adult gut flora is stable and resilient, the biodiversity indices might be not changed by relatively low-intensity or short periods of exercise interventions [26]. Comparing the effects of medium- and low-strength versus high-intensity exercise on GM, the latter was found to induce more marked effects on microbial diversity than the former. A significant higher alpha diversity, represented by the Shannon index, was found by Bielik [24], who asked enrolled athletes to complete a 7-week high-intensity training program. A similar finding was provided by Torquati [15]. Furthermore, the type of intervention was also an important factor in the association with the microbial diversity index [29]. According to the results of this review, the coordinated intervention of varied exercise patterns showed a significant effect on the microbial diversity index of adults [20,21]. Hence, people participating in various sports events might gain a higher flora diversity than those performing a simplex pattern of exercise intervention. Moreover, the relative abundance and community structure of gut flora were found to change in some cases, although no statistical differences in microbial diversity parameters were found [17]. As reported by Wang [46], the gene count, alpha, and beta diversity did not significantly change after a 12-week moderate aerobic exercise in young adolescents. However, the dominant gut flora that was associated did increase after the exercise intervention. It is worth noting that greater alpha diversity of gut flora in adults is not always associated with gut health but might result from the growth of potentially harmful microbial [39,47]. Thus, it is important to explore biodiversity indices and interpret them in the context of specific GM composition changes.

### 4.3. Changes in Specific Flora

As reported by the findings in this review, clear differences in GM compositions among athletes and active and inactive adults were observed at the phylum and genus levels. In line with these studies, we found higher abundances of various microbial in active adults. At the phylum level, Actinobacteria, Bacteroidetes, Firmicutes, Proteobacteria, Verrucomicrobia, and Cyanobacteria are observed to undergo significant changes in most studies. Simultaneously, at the genus level, *Bifidobacterium*, *Anaerostipes*, *Akkermansia*, *Lactococcus,* and *Romboutsia* in exercising adults showed a higher relative abundance in most cases. In contrast, the abundance of *Bacteroides*, *Parabacteroides,* and *Streptococcus* were consistently lower after exercise intervention. As one area where physical exercise might show beneficial effects is the intestinal microbial composition, the analysis of specific flora in the adult gut should be an essential component of reviews of the associations between GM communities and human health.

#### 4.3.1. The Two Most Abundant Phyla: Firmicutes and Bacteroidetes

According to previous studies, Firmicutes and Bacteroidetes are the two most abundant phyla that account for up to 90% of the gut flora in adults. Eight out of the ten studies that mentioned the above two phyla showed an increase in Firmicutes and a decrease in Bacteroidetes in terms of their relative abundance under exercise intervention. The Firmicutes phyla contain various genera of highly diverse bacteria that are beneficial for improving the gut health of adults, while the higher abundance of Bacteroidetes is often associated with poorer microbial composition and certain unhealthy habits such as sedentary behavior and a high-fat diet. However, some genera derived from Bacteroidetes phyla have been suggested to be negatively associated with type 2 diabetes in humans and regarded as the producers of propionate and acetate. Furthermore, the ratio of F/B is usually proposed as an eventual biomarker in research on human gut flora, resulting from its high sensibility in the intervention of physical exercise or other environmental factors. Previous studies frequently regard the high ratio of F/B as a dysbiosis marker in adults, especially within obesity. Although our results showed a slight increase in the F/B after exercise intervention, physical exercise provided a positive function to modulate microbial ecology and enhance gut health. This is likely explained by an elevated protein intake for elite athletes and the high variability of relative abundance between different individuals and metabolic characteristics. Currently, the change in the F/B ratio is not enough to regard exercise interventions as the golden standard of health-producing gut flora regulation. Therefore, it is necessary to modulate the characterization of the subjects and eliminate the influence of co-variables that would interfere with the discussion for results. To sum up, the function of genera belonging to Firmicutes and Bacteroidetes phyla usually shows heterogeneity, and its impact on the human gut following exercise intervention deserves further research.

#### 4.3.2. SCFAs-Producing Bacteria

Intestinal flora plays a pivotal role in the regulation of metabolic, endocrine, and immune functions through their metabolites, and short-chain fatty acids (SCFAs) including acetate, propionate, and butyrate are the main bacterial metabolites in the human gut [48]. SCFAs are commonly provided for building the associations between the microbial composition and health outcomes in humans, which have been declared to maintain the integrity of colonocytes, enhance the barrier function, and increase mucin expression via regulation of the expression of specific genes [49]. Consistent with other studies, the increase in the SCFA concentration or the gene encoding SCFA in actively exercising adults was found in this review, which was attributed to an increase in specific SCFA-producing bacteria in the gut. At the genus level, *Ruminococcus gauvreauii*, *Anaerostipes,* and an uncultured genus from *Lachnospiraceae*, all belonging to the SCFA-producing Firmicutes phylum, were found to have a significantly increasing relative abundance in the adult gut after exercise intervention, which was positively correlated with insulin sensitivity and cardiorespiratory fitness [22,29]. A sharp increase in the abundance of Bifidobacterium was reported in many studies of this review [15,17,26,29,34,38], portending a positive association with producing acetate [50,51]. The metabolic pathway of acetate in the gut epithelial cells could increase the release of IL-18 and then engage the epithelial IL-18 receptor and promote intestinal barrier integrity [52]. The genera *Roseburia hominis* and *Akkermansia muciniphila* were found to have significantly higher relative abundance in actively exercising adults compared to sedentary people [32], and both of these genera were demonstrated to have a beneficial impact on the host gastrointestinal tract health, lipid metabolism, and immune system as they produce butyrate. Likewise, the acetate and butyrate producers represented by *Bifidobacterium* and *Akkermansia muciniphila* were found to significantly increase following an 8-week training intervention by Torquati [15]. In addition, there is enough evidence to support the notion that SCFAs could mediate the potential effects on glucose metabolism. The genus *Dorea*, which produces acetate as one of the main end products of fermentation, was found to be directly associated with glucose in a previous study. In this review, *Dorea* was significantly higher in exercising adults than in their inactive peers [17,29], therefore indicating a beneficial effect in modulating glucose metabolism via an exercise intervention. These results suggested that physical activity and exercise training were helpful for solidifying gut barrier function and regulating microbial metabolism in adults.

#### 4.3.3. Other Representative Bacteria

In this review, the abundance of some representative beneficial bacteria showed a significant difference in athletes compared to sedentary people or other inactive groups, indicating an enhancement of the GM composition and human health. Of these bacteria, the genera of *Bifidobacterium*, *Akkermansia*, *Lactococcus,* and *Coprococcus* were found to have a much higher relative abundance in physically active adults [15,17,20,23,24,26,29,38]. In recent years, *Bifidobacterium* has been widely used as probiotics and declared to have a beneficial impact on many pathological conditions, simultaneously being regarded as the mediator of lactate metabolism for producing butyrate [15,53]. When taken as probiotics, *Bifidobacterium* has been demonstrated to prevent inflammation, decrease the risk of cardiovascular disease, and furthermore, modulate local immune responses and even systemic health in (insert population) [26,54]. Moreover, *Bifidobacterium* could achieve emotional regulation through the brain–gut axis, showing a negative correlation with severe depression in mice [26,55]. Four studies in this review observed a significant difference between groups in *Akkermansia* [14,29,38,39], which has been previously described to have a high prevalence in the gut microbiome of athletes. It is noteworthy that the abundance of *Akkermansia* has been reported to inversely correlate with obesity and metabolic disorders in mice and humans (reference). In agreement with other studies, we observed a positive response in *Akkermansia* abundance to exercise intervention. *Lactobacillus* was another genus that showed sharp enrichment after a high-intensity exercise intervention [24]. As mentioned by Shelton [56], *Lactobacillus* and its derived metabolites could participate in the regulation of lipid metabolism in intestinal epithelial cells and contribute to the prevention of obesity related to a high-fat diet in early life. It was also reported [57] that all nutrient-rich feed and food environments, as well as animal and human mucosae, include *Lactobacillus*. Some species belonging to *Lactobacillus* could convert polyunsaturated fatty acids into health-beneficial fatty acid metabolites [58,59]. Thus, the increasing abundance of *Lactobacillus* induced by physical exercise was suggested to be a promising pathway for improving human metabolism. *Coprococcus*, a butyrate-producing genus that can decrease the severity of atopic disease, was also observed to have noteworthy enrichment among actively exercising adults [20,23,24]. It further demonstrates the health-promoting effects of exercise, indicating the worth of investigating specific gut genera composition in an actively exercising population.

Physical exercise could therefore modulate the gut flora composition towards a healthier state not only by enriching beneficial bacteria but also by decreasing the abundance of pathogenic species. Shin discussed the relationship between the Proteobacteria phylum and the gut flora ecological balance [60], declaring that Proteobacteria could be regarded as a marker of disorder in the GM community and a potential diagnostic criterion for diseases. Here, the abundance of Proteobacteria phyla displayed a reduction in six studies among the seven studies that mentioned it [14,18,21,29,33,37,39]. However, a slight increase in Proteobacteria was detected in elite athletes engaging in strenuous exercise, which might result from the negative impact on the GM because of overloaded or prolonged training. Similarly, several taxa like *Holdemanella* and some of the *Ruminococcus* genus significantly decreased in abundance after an exercise intervention of approximately 2 months [14,26]. *Holdemanella* was usually associated with an unhealthy lifestyle, showing a positive relationship with chronic kidney disease [26,61]. *Ruminococcus* is heterogeneous, simultaneously incorporating beneficial and harmful species while promoting an inflammatory response in most cases.

Given the evidence of gut microbial modulation related to exercise and physical activity in adults, this review attempts to summarize the specific changes in the microbiota diversity index and the abundance of key functional bacteria. It provides a more accurate and intuitive view of academic and clinical research on the health-promoting effect of exercise mediated by intestinal microbiota.

### 4.4. Limitations

Several limitations of this systematic review should be considered. First of all, the diet contribution was not separated from the exercise intervention, while a dietary intervention was one of the exclusion criteria. As one of the known factors influencing the microbial composition in the human gut, researchers cannot afford to ignore diet when discussing the regulation of intestinal flora. Simultaneously, actively exercising people, especially professional athletes, are usually accompanied by healthier dietary patterns, which will provide a potential impact on the process of altering GM. Furthermore, the second limitation comes from the search process; although we proceeded in the process using the PRISMA protocol, there was one article that was sourced from the citations. This is why there is a small possibility that we missed more than one manuscript. The third limitation is the different sequencing regions (V1, V2, V3, and V4), although we limited sequencing analysis methods to 16S rRNA, which could also influence the data in our systematic review. Moreover, some authors were reluctant to share data. Lastly, the selected studies had a wide range of ages. In general, there are differences in the distribution of GM according to age and area. Given that the environment, along with dietary and cultural habits, influences the GM composition, the inclusion of studies from all over the world could be why the between-study heterogeneity values we obtained were so high. Despite these limitations, we systematically searched all raw sequencing data and meta-data and analyzed them in a suitable and uniform manner to minimize heterogeneity.

### 4.5. Future Direction

We have growing knowledge of the effects of exercise on GM, but more mechanism research regarding the physiological communication between the gut and muscles is needed before we can fully understand the interplay between them. Future studies should look into several environmental factors that could affect the gut bacterial composition, including lifestyle, diet, exposure to environmental chemicals, and the use of antibiotics, pre-, or probiotics. In this field, well-designed, randomized exercise intervention studies are needed to assess the therapeutic potential of exercise in the context of GM. Notably, the combined analysis of metabolomic and metagenomic data is considered to provide a new perspective on the synergistic effect of sports, diet, and other environmental factors. The scope may also need to be broadened to evaluate more taxa than those considered here, including the eukaryote (e.g., fungi) members of the GM. Moreover, besides focusing on the specific bacteria in the human gut, prospective studies should provide more evidence of the expression of health-promoting functional genes related to exercise. Micro-level results would provide many more clinical references for the intestinal microbiota modulation induced by exercise and PA. With further exploration, the alternation of GM related to exercise intervention was found to be heterogeneous among different genders and ages. Thus, the different effects among various sports events and longer-duration studies need to be further demonstrated, and coordinated interventions gathering different exercise patterns similarly warrant more attention in the future.

## 5. Conclusions

The present meta-analysis reported a significant increase in the gut microbiota diversity after exercise in adults, particularly reflected in the Shannon Index. When compared to control groups, those who participated in an exercise intervention showed a greater abundance of Firmicutes genera along with a lower abundance of Bacteroidetes. However, researchers should interpret their findings within the consideration of demographic factors and exercise intensity since females and older adults appear to be more sensitive. Therefore, better-designed and longer-duration studies are needed to better assess the chronic effect of exercise on gut microbiota.

## Figures and Tables

**Table 1:** Characteristics of the selected studies.

No.	Authors	Country	Sample (E/C)	Subjects Age (Years)	Sources of Participants	Gender	Exercise Protocol	Control Group	Main Outcomes	Region
1	S. Donati Zeppa et al., 2021 [17]	Italy	17	22 ± 2	healthy college students	both	36 indoor cycling training sessions during nine wks were performed.	self	Alpha diversity indexes (Chao1, Shannon and Simpson)	V3–V4
2	Resende et al., 2021 [18]	Brazil	12	25.18 ± 4.66	healthy Brazilian men	male	3 supervised training sessions per wk on non-consecutive days, lasting 50 min each, were performed over 10 wks	self	Alpha diversity index (Shannon)	V4
3	Laura Mancin et al., 2022 [19]	Turkey	16	25.5 ± 2.8	semiprofessional soccer players	male	8 h of training/wk	self	OTU & Shannon	V3–V4
4	Nazareth Castellanos et al., 2019 [20]	Spain	109 (64 + 45)	32.17 ± 7.40	Universidad Europea de Madrid	both	3 days of exercise per wk for 30 min at a moderate intensity (bicycling at a regular pace, swimming or other fitness activities)	no intervention	Alpha diversity indexes (Chao1, Shannon and Simpson)	V3–V4
5	Fei Zhong et al., 2022 [21]	China	8	66.38 ± 4.07	community	female	4 times per wk. Each session lasted approximately 60 min of combined aerobic exercise (20 min) and resistance exercise (25 min)	self	Alpha diversity indexes (Chao1, Shannon and Simpson)	V3–V4
6	Rebecca J. H. M. Verheggen et al., 2021 [22]	Netherlands	14	51 ± 11	inactive participants with obesity	50%	8 wks’ cycling exercise on an ergometer (Lode), starting with a 5-min warm-up, followed by 50 min of exercise at 65% to 85% of the individual HRR, and ending with a cooldown of 5 min.	self	Alpha diversity indexes (Chao1, Shannon and Simpson)	V5–V6
7	Lucas Moitinho-Silva et al., 2021 [23]	Germany	24 (13 + 11)	30 ± 9.9	healthy physically inactive German male and female	both	run 3 times per wk for at least 30 min for 6 wks	con	Alpha diversity indexes (Chao1, Shannon and Simpson)	V1–V2
8	Siobhan F Clarke et al., 2014 [14]	Ireland	63 (40 + 23)	29 ± 4	Male elite professional rugby players	male	NA	no intervention	Alpha diversity indexes (Chao1, Shannon and Simpson)	V4
9	L. Torquati et al., 2022 [15]	Australia	12	63.29 ± 6.625	T2D	both	HIIT or MICT for 8 weeks	self	Alpha diversity indexes (Chao1, Shannon and Simpson)	NA
10	Viktor Bielik et al., 2022 [24]	Slovakia	12	16–25	swimmers competitive at the national level. The athletes came from two swimming clubs.	both	HIIT for 7 wks	self	Alpha diversity indexes (Chao1, Shannon and Simpson)	V1–V3
11	Elizabeth A Rettedal et al., 2020 [25]	New Zealand	29	20–45	lean and overweight men	male	nine sessions of cycle ergometer HIIT on non-consecutive days over 3 wks	self	Alpha and Beta diversity	V3–V4
12	YeonGyun Jung et al., 2020 [26]	Korea	104	N/A	navy trainees	both	through basic military training, and cardio and weight training performed together every day for 8 wks	self	Alpha and Beta diversity	V4–V5
13	Mariangela Tabone et al., 2021 [27]	Spain	40	35.79 ± 8.01	different cross-country athletes’ teams in Madrid, Spain	male	ran with a slope of 1% at a speed of 10 km/h, with increments of 0.3 km/h every 30 s until volitional exhaustion.	self	Alpha and Beta diversity	V3–V4
14	Owen Cronin et al., 2020 [28]	Ireland	52 (25 + 27)	35 (28, 38)	physically inactive for at least 3 months prior	both	an 8-wk (3 times per wk) mixed aerobic and resistance exercise training program with protein supplementation	protein supplementation	Alpha diversity indexes (Chao1, Shannon and Simpson)	NA
15	Eveliina Munukka et al., 2018 [29]	Finland	17	36.8 ± 3.9	sedentary overweight women	female	a 6 wks endurance exercise	self	Alpha diversity indexes (Chao1, Shannon and Simpson)	V4
16	Shaodong Wei et al., 2022 [30]	Danish	86 (60 + 26)	54.3 ± 8.9	above 18 years old, T2D diagnosis for less than 10 years	both	five to six weekly aerobic sessions whereof two to three sessions were combined with resistance training; 12 months	standard care group	Alpha and Beta diversity	V3–V4
17	C. J. Stewart et al., 2017 [31]	UK	20 (10 + 10)	26.9 ± 5.06	a duration of diabetes > 5 years	male	aerobic-based exercise for a minimum of 30 min at a time, at least three times per week	self		V4
18	Carlo Bressa et al., 2017 [32]	Spain	40 (19 + 21)	30.7 ± 5.9	premenopausal women	female	at least 3 h of physical exercise per week	sedentary women	Alpha diversity indexes (Chao1, Shannon H and Simpson)	V3–V4
19	Xueqing Zhang et al., 2023 [33]	China	93	20.23 ± 0.62	College Students with IDA	both	8 wk of Tai Chi, 3 times a wk, 60 min, or 8-week conventional exercise, 60 min, 3 times per wk	no intervention	Alpha diversity (Shannon, Chao1, Simpson) & Beta diversity	V3–V4
20	Camilla J Williams et al., 2022 [34]	Australia	40 (20 + 20)	30.4 ± 9.8	sedentary and apparently healthy adults	both	6 wk of supervised HIIT (4 × 4-min bouts at 85–95% HRmax, 3·wk^–1^)	self	Alpha diversity indexes (Shannon)	NA
21	Runtan Cheng et al., 2022 [35]	China	40 (22 + 18)	59.4 ± 0.5	nonalcoholic fatty liver disease	both	progressive aerobic exercise training program, 2–3 times a wk, 30–60 min per session with 60–75% of the VO2Max.	no intervention	Alpha diversity indexes (Shannon)	V3–V4
22	Kristen S.Smith et al., 2022 [36]	USA	24	20.8 ± 1.7	young female adults	female	10 wks of Resistance T raining	self	Alpha diversity indexes (Shannon and Simpson)	V4
23	Dongyang Kang et al., 2023 [37]	China	30 (15 + 15)	N/A	basketball players from university	male	90 min Tai Chi, 6 days per week, 5 months	no intervention	OUT & Alpha diversity indexes (Chao1, Shannon, Faith’s PD)	V3–V4
24	Yuping Zhu et al., 2023 [38]	China	32	30.30 ± 5.28	MA dependent individuals	both	aerobic exercise, 3 times per wk, 60 min, 8 wks.	self	Alpha diversity indexes (Shannon)	V3–V4
25	Shrushti Shah et al., 2023 [39]	Canada	110	56.23 ± 6.36	People with normal BMI and High BMI	both	IPAQ	people with low physical activity	Alpha diversity indexes (Shannon) & Beta diversity (Bray–Curtis dissimilarity metrics)	V3–V4

BMI: body mass index; wk: week/s; VO2max: maxima oxygen uptake; HRR: heart rate reserve; HIIT: high-intensity interval training; MICT: Moderate Intensity Continuous Training; T2D: type 2 diabetes; HRmax: maximal heart rate; N/A: Not Applicable; MA: methamphetamine, IPAQ: International Physical Activity Questionnaire; OTU: Operational Taxonomic Units; IDA: Internet addiction disorder.

## Data Availability

Data sharing is not applicable to this study, as no new data were created. However, the authors confirm that the data supporting the findings of this study are available within the article, its Supplementary Materials, and referenced publications from which the data were extracted.

## Supplementary Materials

The following supporting information can be downloaded at: https://www.mdpi.com/article/10.3390/nu16071070/s1, Figure S1: Risk of bias graph: review authors’ judgements about each risk of bias item presented as percentages across all included studies; Figure S2: Risk of bias summary: review authors’ judgements about each risk of bias item for each included study; Figure S3: Funnel Plot for Shannon Index; Figure S4: Funnel Plot for Simpson Index; Figure S5: Funnel Plot for Observed OUT; Figure S6: Funnel Plot for Chao1 Index; Figure S7: Funnel Plot for PD Index.
