# Supplementary material for: Effects of Exercise on Gut Microbiota of Adults: A Systematic Review and Meta-Analysis
Source: Nutrients. 2024 Apr 5;16(7):1070. doi: 10.3390/nu16071070 (PMC11013040; doi:10.3390/nu16071070)
Supplement: Supplementary file 1 [file nutrients-16-01070-s001.zip › nutrients-2907963-supplementary.pdf]

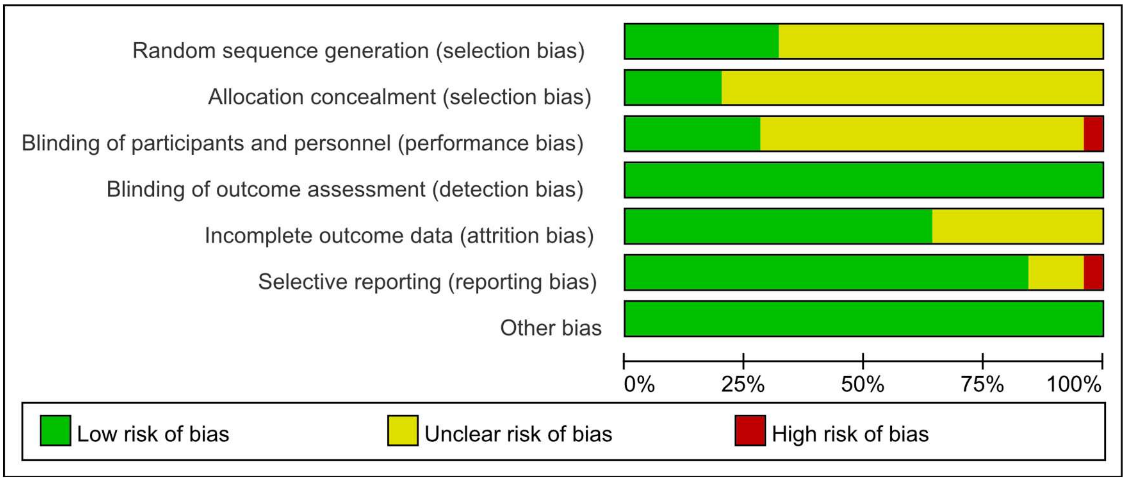

Figure S1 Risk of bias graph: review authors' judgements about each risk of bias item presented as percentages across all included studies.

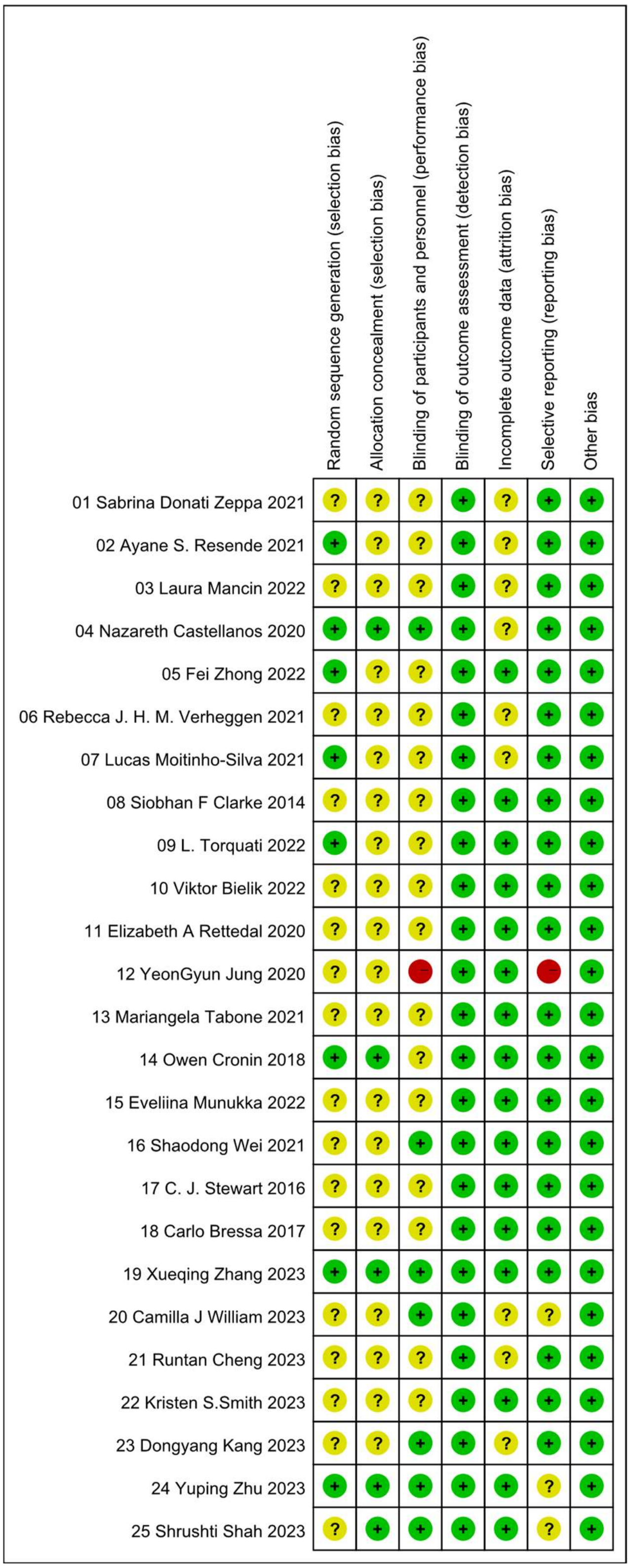

Figure S2 Risk of bias summary: review authors' judgements about each risk of bias item for each included study.

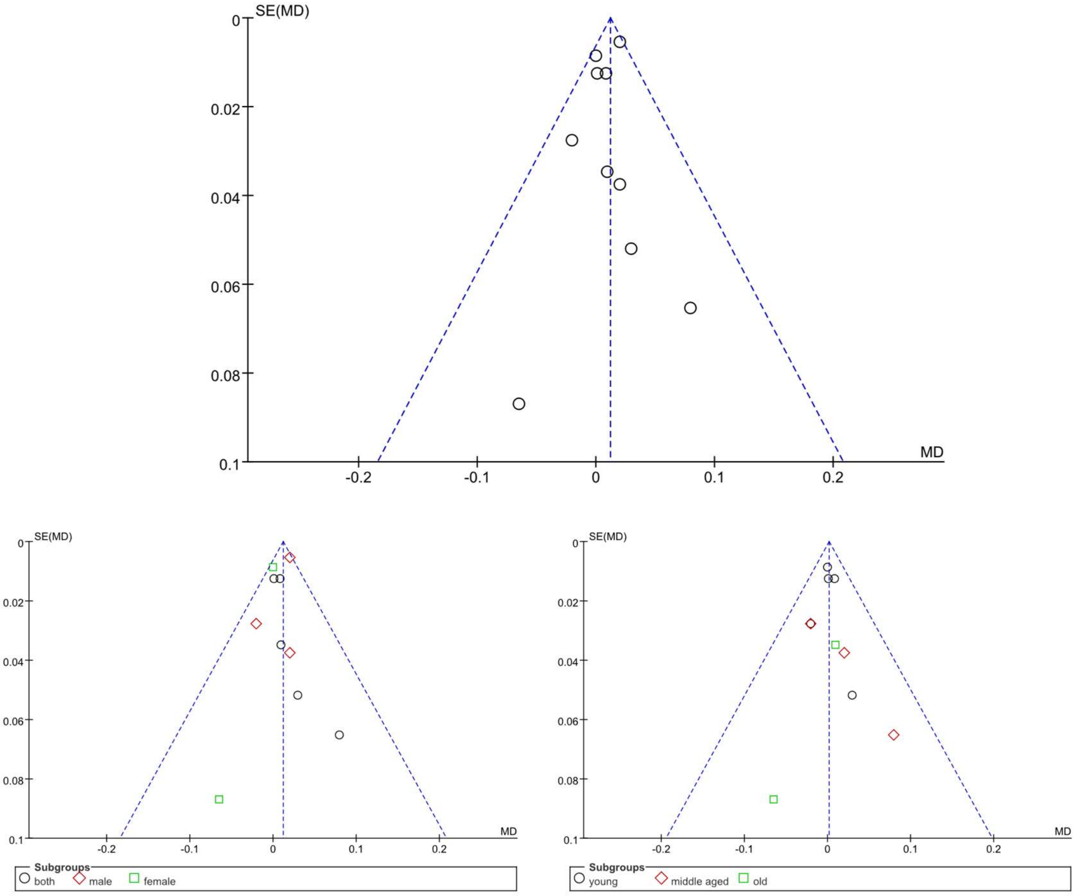

Figure S3 Funnel Plot for Shannon Index

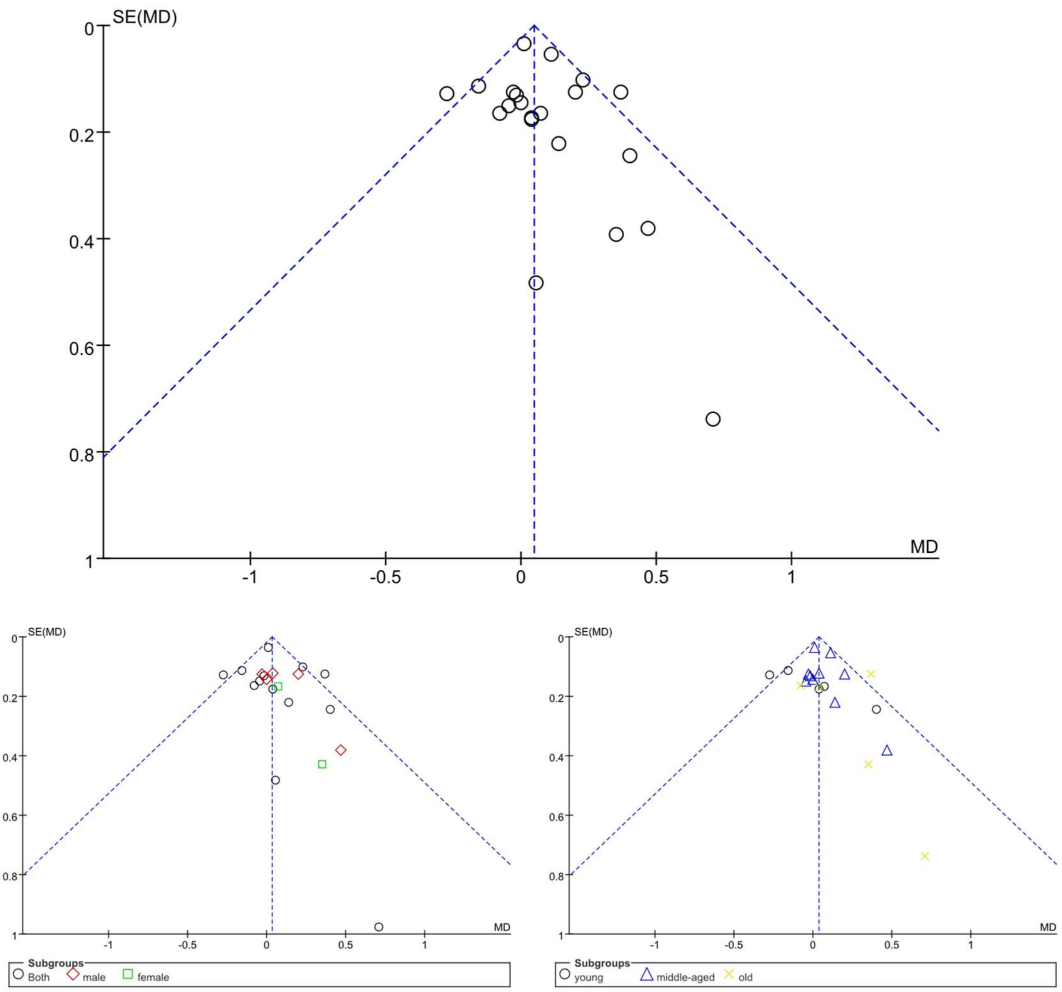

Figure S4 Funnel Plot for Simpson Index

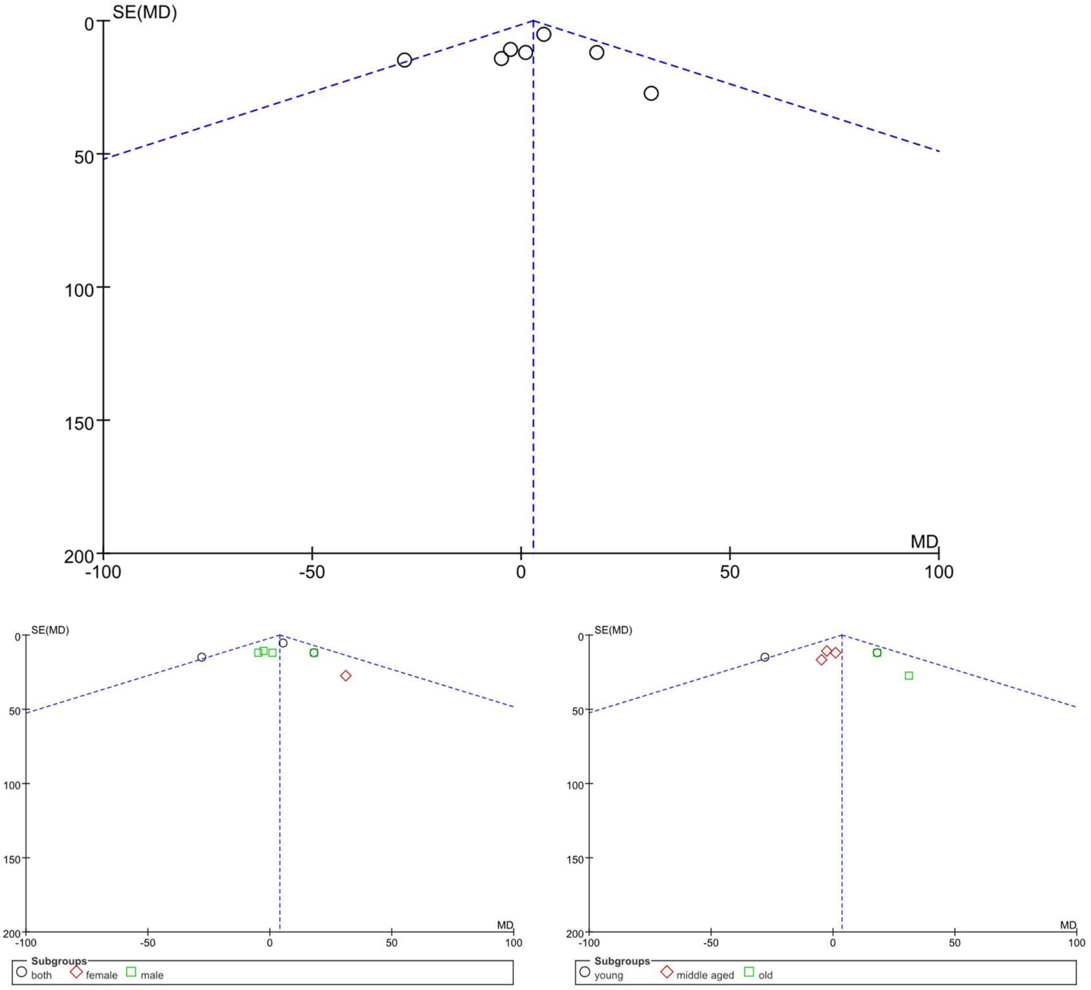

Figure S5 Funnel Plot for Observed OUT

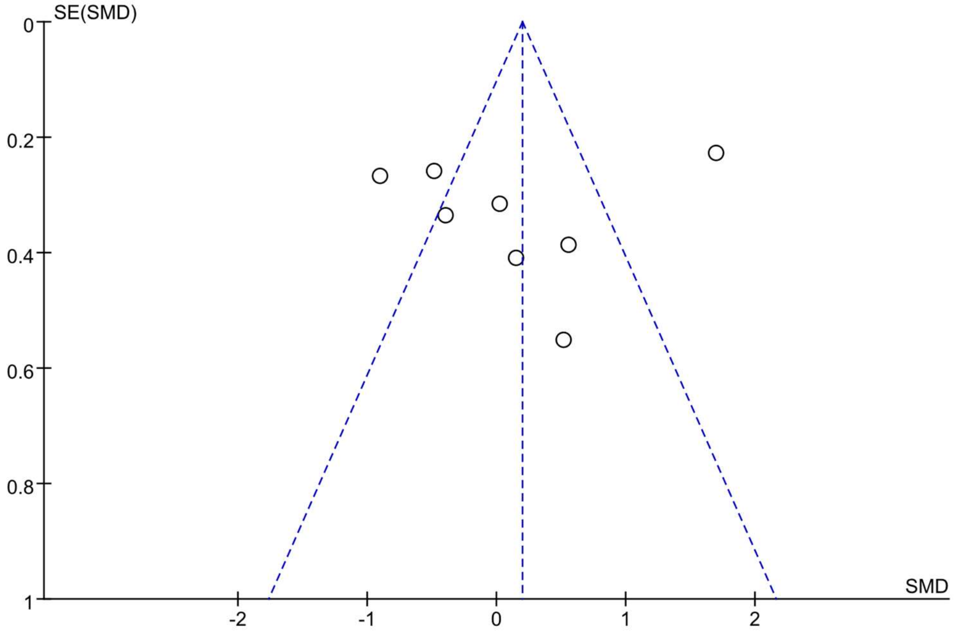

Figure S6 Funnel Plot for Chao1 Index

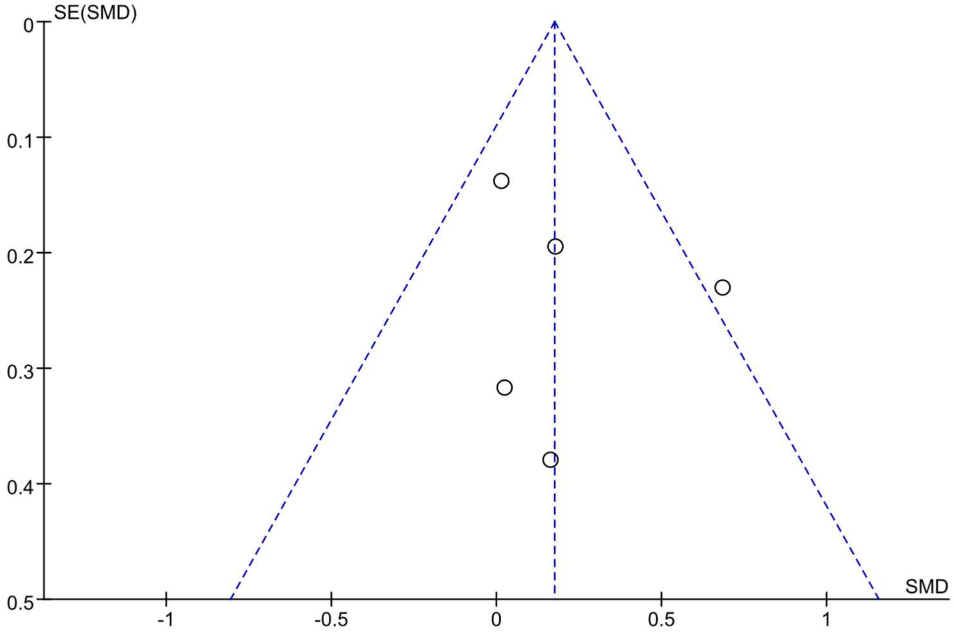

Figure S7 Funnel Plot for PD Index
